# The Vsr-like protein FASTKD4 regulates the stability and polyadenylation of the *MT-ND3* mRNA

**DOI:** 10.1093/nar/gkae1261

**Published:** 2024-12-27

**Authors:** Xuan Yang, Maike Stentenbach, Laetitia A Hughes, Stefan J Siira, Kelvin Lau, Michael Hothorn, Jean-Claude Martinou, Oliver Rackham, Aleksandra Filipovska

**Affiliations:** Department of Molecular Cell Biology, University of Geneva, Quai Ernest-Ansermet 30, 1211 Geneva, Switzerland; The Kids Research Institute Australia, Northern Entrance, Perth Children's Hospital, 15 Hospital Avenue, Nedlands, Western Australia 6009, Australia; ARC Centre of Excellence in Synthetic Biology, 15 Hospital Avenue, Nedlands, Western Australia 6009, Australia; The Kids Research Institute Australia, Northern Entrance, Perth Children's Hospital, 15 Hospital Avenue, Nedlands, Western Australia 6009, Australia; ARC Centre of Excellence in Synthetic Biology, 15 Hospital Avenue, Nedlands, Western Australia 6009, Australia; The Kids Research Institute Australia, Northern Entrance, Perth Children's Hospital, 15 Hospital Avenue, Nedlands, Western Australia 6009, Australia; ARC Centre of Excellence in Synthetic Biology, 15 Hospital Avenue, Nedlands, Western Australia 6009, Australia; Department of Plant Sciences, University of Geneva, Quai Ernest-Ansermet 30, 1211 Geneva, Switzerland; Department of Plant Sciences, University of Geneva, Quai Ernest-Ansermet 30, 1211 Geneva, Switzerland; Department of Molecular Cell Biology, University of Geneva, Quai Ernest-Ansermet 30, 1211 Geneva, Switzerland; The Kids Research Institute Australia, Northern Entrance, Perth Children's Hospital, 15 Hospital Avenue, Nedlands, Western Australia 6009, Australia; ARC Centre of Excellence in Synthetic Biology, 15 Hospital Avenue, Nedlands, Western Australia 6009, Australia; Curtin Medical School, Curtin University, Kent St, Bentley, Western Australia 6102, Australia; Curtin Health Innovation Research Institute, Curtin University, Kent St, Bentley, Western Australia 6102, Australia; The Kids Research Institute Australia, Northern Entrance, Perth Children's Hospital, 15 Hospital Avenue, Nedlands, Western Australia 6009, Australia; ARC Centre of Excellence in Synthetic Biology, 15 Hospital Avenue, Nedlands, Western Australia 6009, Australia; Curtin Medical School, Curtin University, Kent St, Bentley, Western Australia 6102, Australia

## Abstract

Expression of the compact mitochondrial genome is regulated by nuclear encoded, mitochondrially localized RNA-binding proteins (RBPs). RBPs regulate the lifecycles of mitochondrial RNAs from transcription to degradation by mediating RNA processing, maturation, stability and translation. The Fas-activated serine/threonine kinase (FASTK) family of RBPs has been shown to regulate and fine-tune discrete aspects of mitochondrial gene expression. Although the roles of specific targets of FASTK proteins have been elucidated, the molecular mechanisms of FASTK proteins in mitochondrial RNA metabolism remain unclear. Therefore, we resolved the structure of FASTKD4 at atomic level that includes the RAP domain and the two FAST motifs, creating a positively charged cavity resembling that of the very short patch repair endonuclease. Our biochemical studies show that FASTKD4 binds the canonical poly(A) tail of *MT-ND3* enabling its maturation and translation. The *in vitro* role of FASTKD4 is consistent with its loss in cells that results in decreased *MT-ND3* polyadenylation, which destabilizes this messenger RNA in mitochondria.

## Graphical Abstract

**Graphical Abstract:**
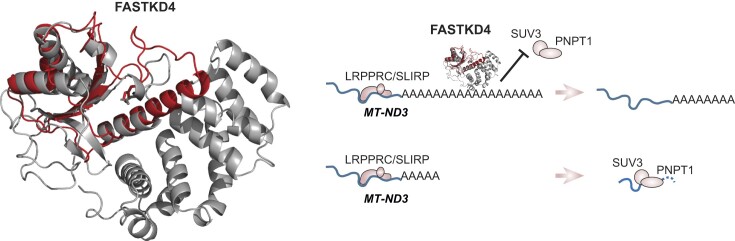

## Introduction

Mammalian mitochondrial gene expression is predominantly regulated by RNA-binding proteins (RBPs) that are nuclear encoded and post-transcriptionally imported into mitochondria (1). The unique regulation of mitochondrial DNA (mtDNA) is a consequence of its small and compact structure that has been depleted of non-coding regions (2). Transcription of mtDNA generates genome-wide long polycistronic transcripts (3) that are processed into messenger RNAs (mRNAs), transfer RNAs (tRNAs) and ribosomal RNAs (rRNAs) by the proteinaceous RNAse P (PRORP) that is part of the RNase P complex (4,5) and ELAC2 (6). The 11 mitochondrial mRNAs (mt-mRNAs), devoid of 5′ untranslated regions, are translated on mitochondrial ribosomes (mitoribosomes) using the full complement of 22 mtDNA-encoded tRNAs required for protein translation (1). Mitoribosomes are composed of two mtDNA-encoded rRNAs and over 80 nuclear encoded, mitochondrially targeted ribosomal proteins that are essential for the translation of mt-mRNAs (1). Diverse RBPs modulate the stability, modification, translation and turnover of mt-RNAs, including the pentatricopeptide repeat (7) and Fas-activated serine/threonine kinase (FASTK) protein families (8).

Members of the FASTK families share the RNA-binding domains FAST1, FAST2 and RAP, the latter of which is known for its nuclear and chloroplast nuclease activity for RNA splicing and processing (8). Consequently, some of the FASTK members have roles in mt-RNA processing of the non-canonical junctions that are not spanned by tRNAs, such as FASTKD5 that processes *MT-ND5:MT-CYTB* and *MT-ATP8/6:MT-CO3* (9,10) or FASTK that is required for pre-*ND6* cleavage (8). The FASTKD2 protein has a role in mtRNA stability and translation and mutations in its gene cause mitochondrial disease (9,11–15). However, the roles of the remaining FASTKD proteins, such as FASTKD4, remain less well understood.

Recently, we showed that loss of FASTKD4 led to reduced stability of specific mt-mRNAs and non-coding mtRNAs, affected the processing of the *MT-ND5:MT-CYTB* junction, possibly through its association with tRNA^E^ and decreased translation (10). To understand the molecular role of FASTKD4 in mitochondrial gene expression we resolved the RAP domain and FAST1 and FAST2 components of its structure at an atomic level, revealing its architecture. Here we identify that FASTKD4 is required for *MT-ND3* maturation and stability, and show that loss of FASTKD4 decreases mt-mRNA polyadenylation in mitochondria.

## Materials and methods

### Cell culture

HAP1 cells were cultured and maintained at 37°C under humidified 95% air/ 5% CO_2_ in Iscove's Modified Dulbecco's Medium supplemented with 4 mM L-glutamine, 25 mM HEPES, 3.024 g/l NaHCO_3_, 10% fetal bovine serum (FBS), 100 U/ml penicillin, 100 mg/ml streptomycin, 1 mM sodium pyruvate and 50 μg/ml uridine. 143B cells, CAL51, HeLa, 143B and HEK293T cells were grown in Dulbecco's Modified Eagle Medium supplemented with 4 mM L-glutamine, 10% FBS, 100 U/ml penicillin, 100 mg/ml streptomycin, 1 mM sodium pyruvate and 50 μg/ml uridine.

### Generation stable knockout cell lines

HAP1, CAL51 and 143B cells were seeded at 60% confluency 24 h prior to transfection. Cells were transfected with 1.5 μg of a pD1311-AD mammalian Cas9 expression vector bearing a *FASTKD4*-targeting gRNA using FuGENE HD (Promega) in Opti-MEM. Additionally, HAP1 wild-type (WT) and FASTKD4 knockout cells were transfected with either an *ANGEL2*- or *NOCT*-targeting gRNA. Single cells were sorted into 96-well plates based on GFP fluorescence signal using a FACSAria II (BD Biosciences) in PBS supplemented with 2% FBS 72 h post transfection. Sanger sequencing was performed by the Australian Genomic Research Facility (AGRF) to confirm the introduction of frameshift-inducing deletions in *FASTKD4*, *ANGEL2* or *NOCT* genes.

### RNA extraction and northern blotting

HAP1 or CAL51 WT or knockout cells were treated with 20 μg/ml chloramphenicol 24 h prior to RNA extraction. RNA from treated or untreated cells was extracted using the miRNeasy Mini kit (Qiagen) incorporating an on-column RNase-free DNase digestion to remove all DNA. Northern blotting was performed as described previously (16). Four microgram to 15 μg RNA (depending on the experiment) was resolved on 1.2% agarose formaldehyde gels, transferred to a 0.45-μm Hybond-N^+^ nitrocellulose membrane (GE Lifesciences), and hybridized with biotinylated oligonucleotide probes specific to *MT-ND3*, *MT-ND5*, *MT-ND6*, *MT-ATP8/6*, *MT-CO3* and *MT-CYB* mRNAs. Blots were hybridized overnight at 50°C in 5 × SSC, 20 mM Na_2_HPO_4_, 7% SDS and 100 μg ml^−1^ heparin, followed by washing. An 18S rRNA probe was used as a loading control. A streptavidin-linked infrared-labelled antibody [diluted 1:2000 in 3 × SSC, 5% SDS and 25 mM Na_2_HPO_4_ (pH 7.5)] was used to detect the signal on an Odyssey Infrared Imaging System (Li-Cor).

### Poly(A) tail capture PCR and sequencing

The poly(A) length was detected as previously described (17,18). Two and a half micrograms of total RNA was ligated to an adaptor DNA oligonucleotide using T4 RNA ligase (New England Biolabs) for 3 h at 37°C. The ligated RNA was isolated by UltraPure (ThermoFisher) phenol, chloroform and alcohol precipitation followed by reverse transcription using SuperScript III Reverse Transcriptase (Invitrogen) with an anti-adaptor primer. PCR was performed with Phusion Plus DNA polymerase (Thermo Scientific) using a primer designed to bind the ligated adaptor RNA sequence and a gene-specific primer for each RNA target. The primers incorporated Illumina adaptor sequences and PCR products were sequenced using an Illumina MiSeq or via Sanger sequencing at the Australian Genome Research Facility (AGRF, Western Australia).

### Poly(A) tail analysis

All quality control was performed with FastQC (0.12.1) and summarized with MultiQC (1.14) (19). Sequenced reads were trimmed of adapters with Cutadapt (20) (4.4) (-a ATGTGAGATCATGCACAGTCATA -A CTGTCTCTTATACACATCTGACGCTGCCGACGA -G TATGACTGTGCATGATCTCACAT -n 2) and trimmed read pairs merged with BBMerge (21) (39.01), retaining only successfully merged reads. Merged reads containing at least 3 A nucleotides at their 3′ ends were extracted with Cutadapt (-a AAA$ -e 0 -O 3 –action = none) and this subset was subsequently trimmed of polyA sequences (–poly-a –action = trim). Poly(A) trimmed reads were aligned to the hg38 mitochondrial genome sequence with Bowtie2 (22) (2.5.1) using default parameters. Depth files of the 3′ terminal nucleotide positions of the mapped, polyA trimmed reads were produced with Bedtools (23) (2.30.0) (genomecov -d -3 -scale [1000/total mapped reads]) and scaled to reads per 1000 mapped, and formatted into bedgraph files with awk (5.1.0). Samtools (24) (1.17) and awk were used to parse the CIGAR string of the alignments to determine the total mapped length on the reference sequence, which was then used as an offset from the 5′ mapping position to identify the subset of reads where the 3′ end mapped position was located at specific sites (such as the canonical 3′ end sites).

### Protein expression and purification

The truncated cDNA sequence of FASTKD4 (Q309-K631, construct KXH7) was cloned into a modified pET-MBP plasmid via Gibson Assembly. The resulting plasmid, KXH7, was transformed into Rosetta *Escherichia coli* competent cells. Twenty milliliters of overnight culture derived from a single colony was transferred into 800 ml LB medium supplemented with 50 μg/ml kanamycin and incubated at 37°C degrees for 2.5 h until an OD600 of 0.6–0.8 was reached. Then, the bacterial culture was cooled to 4°C degrees followed by IPTG induction at 180 rpm and 16°C degrees for 20 h. Bacteria were collected at 4000 g for 15 min and the bacterial pellet was resuspended in PBS supplemented with 10% glycerol, and frozen at −80°C. Frozen bacteria were thawed in PBS, lysed thoroughly using a French Press, and centrifuged at 20000 g for 10 min. The supernatant was loaded on a PBS washed nickel column followed by washing with PBS containing 20 mM imidazole. Protein was eluted, concentrated and injected into a gel filtration column (HiLoad Superdex 200) and equilibrated in 150 mM KCl, 20 mM HEPES pH 7.8 buffer. The peak fractions were collected and concentrated to ∼33 mg/ml for crystal screening using a Mosquito (SPT Labtech) (Supplementary Figure S1A).

### Protein crystallization and data collection

Orthorhombic crystals of MBP-FASTKD4 (residues 309–631) developed at room temperature in sitting drops composed of 0.2 μl of protein solution (MBP-FASTKD4 at 33 mg/ml in150 mM KCl, 20 mM Hepes pH 7.8) and 0.2 μl crystallization buffer (0.06M KHPO4, 15% PEG8000) suspended over 0.07 ml of the latter as reservoir solution. Crystals were transferred in reservoir solution supplemented with sucrose to a final concentration of 30% (w/v) and snap-frozen in liquid N_2_ (Supplementary Figure S1B). A complete dataset was collected at beamline X06DA at the Swiss Light Source, Villigen, Switzerland (Supplementary Table S1). Data processing and scaling was done with XDS (25).

### Structure solution and refinement

The structure of MBP-FASTKD4 was solved by molecular replacement as implemented in the program Phaser (26), using Protein Data Bank (http://rcsb.org) ID 5B3Z as the initial search model. The structure was completed in iterative rounds of manual model-building in COOT (27) and restrained refinement in phenix.refine (28). Inspection of the final models with phenix.molprobity (29) revealed excellent stereochemistry (Supplementary Table S1).

### *In vitro* RNA-binding assays

Five milligrams of mitochondrial lysates from HEK293T cells were incubated with 4 μg *in vitro* transcribed biotinylated *MT-ND3* RNA [incorporating either a 51 nt poly(A) tail, a 25 nt poly(A) tail, without a poly(A) tail, a flanking tRNA-R sequence or a mirror *MT-ND3* RNA sequence, that would result in the formation of a double stranded version of the *MT-ND3* mRNA] and 50 μl of streptavidin beads overnight on ice. RNA was isolated from the beads as described above. Proteins of the input and bound fractions were resolved by SDS-PAGE.

Purified FASTKD4 was incubated at room temperature for 30 min with fluorescein labelled RNA oligonucleotides (Dharmacon) in 10 mM HEPES (pH 8.0), 1 mM EDTA, 50 mM KCl, 2 mM DTT, 0.1 mg/ml fatty acid-free BSA and 0.02% Tween-20. Binding reactions were analysed by 6% PAGE in TAE and fluorescence was detected using a Typhoon FLA 9500 biomolecular imager (GE). Probes for EMSA were as below:

- **ND3-polyA-RNA**: 5′- FAM/rCrArArArArArGrGrArUrUrArGrArCrUrGrArArCrCrGrArArUrArArArArArArArArArArArArArArArArArArArArArArArArA-3′
- **CO2-polyA-RNA:** 5′- FAM/rCrArCrCrCrCrCrUrCrUrArCrCrCrCrCrUrCrUrArGrArGrCrCrArArArArArArArArArArArArArArArArArArArArArArArArA-3′

where r designates a ribonucleotide and FAM designates a 5′ fluorescein modification.

### Immunoblotting

Proteins were resolved by SDS-PAGE and transferred to PVDF membranes (GE Healthcare). The following primary antibodies were used: anti-FASTKD4 (Proteintech, 16245-1-AP), anti-LRPPRC (Santa Cruz, sc-166178), anti-GRSF1 (Sigma, AV40382), anti-PNPT1 (Santa Cruz, sc-365049) and anti-β-actin (Abcam, ab6276). Proteins were visualized via anti-rabbit or anti-mouse HRP-conjugated secondary antibodies (Dako) and using ECL (Amersham).

### siRNA treatment

143B and HAP1 WT and FASTKD4 knockout cells were transfected with 10 nM luciferase, GRSF1, PNPT1, Suv3, MRPP1 or ELAC2 siRNA for 72 h. RNA was extracted as described above, followed by northern blotting.

### Immunoprecipitation

143B cells were transfected with GFP-tagged FASTKD4 plasmids for 72 h. Cells were lysed in 260 mM sucrose, 100 mM KCl, 20 mM MgCl_2_, 10 mM Tris–HCl, pH 7.5, 1% digitonin and complete EDTA-free protease inhibitor cocktail for 30 min at 4°C and lysates were incubated with GFP-Trap Dynabeads (Chromotek) for 2 h at 4°C. Beads were washed in digitonin-wash buffer (same as lysis buffer except with 0.1% digitonin) followed by washing with dilution buffer (same as lysis buffer without digitonin) twice. Eluates were resolved by SDS-PAGE and electrophoretically transferred to PVDF membranes (GE Healthcare). Following primary antibodies were used: anti-FASTKD4 (Proteintech, 16245-1-AP) and anti-LRPPRC (Santa Cruz, sc-166178). Proteins were visualized via anti-rabbit or anti-mouse HRP-conjugated secondary antibodies (Dako) and using ECL (Amersham). Equal protein loading was confirmed by silver staining.

## Results

### The high-resolution structure of FASTKD4

**Figure 1.**
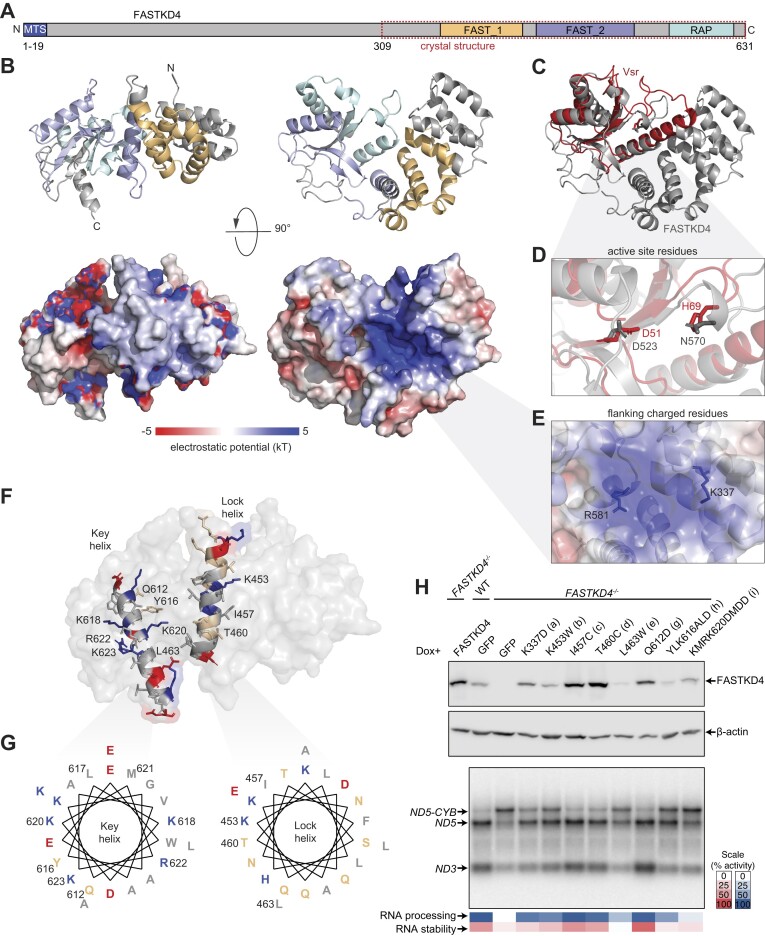
Atomic structure of FASTKD4 and the impact of mutations on its function. (**A**) Schematic structure of FASTKD4 containing an N-terminal mitochondrial targeting sequence and the C-terminal FAST_1, FAST_2 and RAP domains. (**B**) Atomic structure and electrostatic potential of the surface of FASTKD4. (**C**) Structural alignment of FASTKD4 (shown in grey) with the Vsr endonuclease (red). (**D**) The potential catalytic pocket of FASTKD4 in comparison with Vsr. (**E**) Positions of flanking charged residues that contribute to the positively charged cavity. (**F**) Positions of the ‘Lock’ and ‘Key’ helices with key residues indicated. (**G**) Helical wheel projections for the ‘Lock’ and ‘Key’ helices (generated by pepwheel). (**H**) Detection of protein and RNA levels in *FASTKD4* (*D4^−/−^*) mutants targeting either the positively charged surface (K337D and K453W), the central lock helix (I457C, T460C and L4663W) or C-term key helix (Q612D, 616YLKtoALD and 620KMRKtoDMDD). Six micrograms of RNA or 20 μg of protein were separated by size via agarose formaldehyde gel electrophoresis or SDS-PAGE, respectively, transferred to a respective membrane and probed for FASTKD4, β-actin, *MT-ND3* and *MT-ND5*.

The atomic structure of FASTKD4 from residues 309–631, that includes the two FAST and RAP domains (Figure 1A), was determined at 2.15 Å resolution (Figure 1B). The FAST1 domain is composed of three alpha helices, whereas the FAST2 and RAP domains are composed of two alpha helices spanned by two beta sheets and loops (Figure 1B). The FAST2 domain separates the RAP domain from the FAST1 domain, creating a positively charged cavity that could potentially bind negatively charged RNA (Figure 1B). This cavity shares striking similarity to the structures of the very short patch repair (Vsr) and I-Bth0305I bacterial endonucleases (Figure 1C and Supplementary Figure S1C) (30,31), where the Asn in position 570 is juxtaposed to Asp 523 in FASTKD4, compared to the conserved pairing of His 69 to Asp 51 in the active site of Vsr (Figure 1D). The distance between D523 and N570 in FASTKD4 is slightly extended compared to D51 and H69 in Vsr (Supplementary Figure S1D), however, the equivalent of H69 in I-Bth0305I has been shown to adopt a different conformation (31) and active site metal co-ordination is known to be reconfigured upon substrate binding in Vsr (32). The FASTKD4 cavity has an overall positive charge, and this cavity could accommodate a negatively charged RNA substrate (Figure 1E). Another apparent structural feature is the central helix (Q445-E464), that we have referred to as a ‘Lock’, flanked by two unidentified loops without sufficient electron density (Figure 1F and G). The structural similarities between FASTKD4 and the 1VSR and 3R3P orthologues were restricted to the catalytic core of these enzymes and the cavity identified in FASTKD4. The C-terminal helix of FASTKD4, which we have dubbed as ‘Key’, was not aligned well and the lock helix was missing in 1VSR and 3R3P (Supplementary Figure S1B), suggesting a distinct fold in the FASTKD4 structure.

### Mutagenesis of FASTKD4 residues reveals an inactive catalytic site

To identify the potential importance of the key identified features in the FASTKD4 protein, we designed several mutants. Each of the mutants targeted either the charged residues on the surface, the central ‘Lock’ helix or the ‘Key’ C-terminal helix of FASTKD4 (Figure 1F). Since K337, K343, R378, K453 and R581 are the major contributors of the positive charges found in the cavity of FASTKD4, K337D (mutant **a**) and K453W (mutant **b**) were engineered to disrupt this surface. I457C (mutant **c**) and T460C (mutant **d**) on this helix were designed to force a disulfide bond formation with the adjacent C417 and potentially crosslink the central ‘Lock’ helix. L463W (mutant **e**) on this helix was introduced to potentially provide a stronger hydrophobic interaction with Y518 via pi-stacking. Mutants **c**, **d** and **e** were designed to stabilize the central ‘Lock’ helix and to prevent its displacement.

The Q612 residue is exposed at the beginning of the C-term ‘Key’ helix (Figure 1F and G) and because Q612 could be replaced by K, H or V in other species (Supplementary Figure S2), a Q612D (mutant **g**) was introduced to disrupt helix insertion by introducing a negative charge while minimizing the stacking. The Y616A, L617A, K618A and K620A residues in the RAP domain have been mutated previously to show a loss of function (33). Here, Y616 and K618 were mutated from 616YLK to 616ALD (mutant **h**). Y616 can be replaced with F or H, but not W in other species (Supplementary Figure S2). If inserted, Y616 may form pi-stacking with W414 and therefore, the Y616A mutant was designed to prevent this potential interaction. K618 can be replaced with N, Q or R in other species (Supplementary Figure S2), hence K618D was designed to reverse the charge while minimizing stacking. L617 is not exposed to the surface and was not mutated since its mutation could destabilize the observed interaction that stabilized the C-terminal ‘Key’ helix. K620 was mutated together with another two more exposed positively charged residues, R622 and K623. The positive charges were mutated to D, as 620KMRK–620DMDD (mutant **i**).

To assess the activity of the mutants described above, we expressed them in FASTKD4 knockout (*FASTKD4^−/−^*) HeLa cells (34) (Figure 1H). The *FASTKD4^−/−^* cells show a defect in the processing of the *MT-ND5* mRNA, with increased levels of *ND5-CYB* precursor and decreased levels of processed *ND5* mRNA (Figure 1H). Furthermore, these cells show low levels of *MT-ND3* mRNA (Figure 1H). This phenotype can be reverted by expression of the WT FASTKD4 protein. Northern and western blot analyses showed that mutants **a** and **b** had intermediate protein levels with mutant **a** showing higher protein levels, and an increased ability to rescue the processing of *MT-ND5* and the stability of *MT-ND3* mRNAs in *FASTKD4* knockout cells, compared to mutant **b** (Figure 1H). Mutants **e** and **h** had low protein levels and reduced the stability of FASTKD4, which correlated with impaired processing of *MT-ND5* and loss of *MT-ND3* mRNAs (Figure 1H), and their phenotypes were not rescued. These mutants indicate the importance of the ‘Lock’ and ‘Key’ helices for the stability of FASTKD4. Although the stability of the protein was reduced, the amount of protein remaining was proportional to the level of RNA processing observed in the northern blot. This suggested that these mutants affected the stability but not activity of FASTKD4. Mutants **c**, **d** and **g** showed high levels of stable FASTKD4 protein, and their phenotypes were rescued. Mutant **i** had a similar protein amount as the WT protein and mutant **b**, however, the rescue level was very low, which indicated that mutant **i** disrupted the role of FASTKD4 in both the processing of *MT-ND5* and the stability of *MT-ND3* mRNAs (Figure 1H). We mutated the cavity site residue D523 to alanine and found that this mutation did not affect the stability of the protein, and it did not affect the processing of *MT-ND5* nor the stability of *MT-ND3* mRNAs (Supplementary Figure S1E), indicating that FASTKD4 may have lost its endonuclease activity and has been repurposed as an adapter protein for regulating the stability and maturation of specific mitochondrial transcripts. We conclude that L463 and residues 616–618 are required for the stability of FASTKD4, whereas the positively charged residues 620, 622 and 623, and therefore the Key helix interactions, are important for its activity.

### FASTKD4 is necessary for non-canonical RNA processing and *MT-ND3* mRNA stability

**Figure 2.**
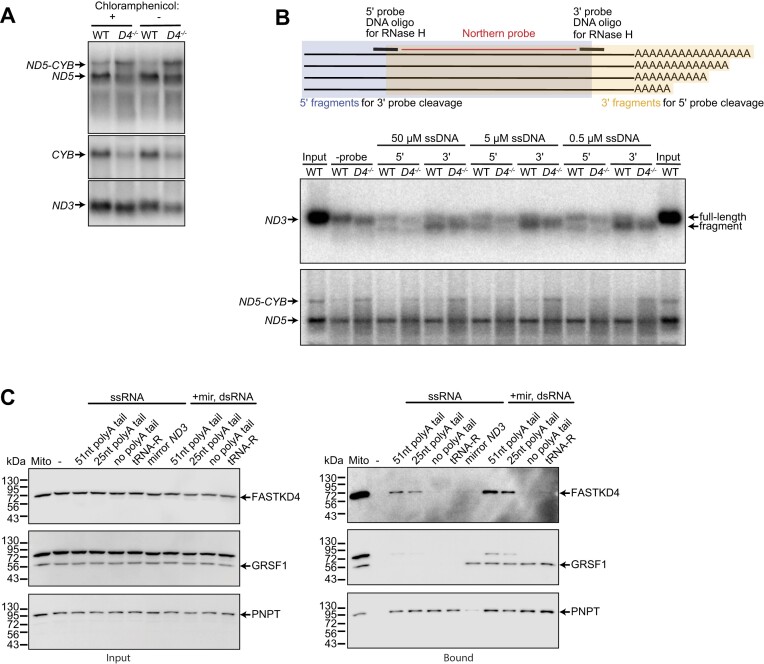
FASTKD4 is required for non-canonical RNA processing and *MT-ND3* mRNA stability. (**A**) Detection of RNA levels through northern blotting after chloramphenicol treatment and in the presence (WT) and absence of FASTKD4 (*D4^−/−^*). Northern blotting was performed using 10 μg of RNA and probed for *MT-ND3*, *MT-ND5* and *MT-CYB* mRNAs. (**B**) Detection of *MT-ND3* mRNA length in WT and *D4^−/−^* cells after incubation with ssDNA (50 μM, 5 μM or 0.5 μM) and RNase H. Northern blotting was performed using 10 μg RNA and probed for *MT-ND3*, *MT-ND5* and *MT-CYB* mRNAs. (**C**) HEK293T mitochondrial lysates were incubated with 4 μg of the following *in vitro* transcribed *MT-ND3* RNAs: 51 nt poly(A) tail, 25 nt poly(A) tail, without poly(A) tail, tRNA-R or mirror *MT-ND3* RNA. Protein levels were detected by SDS-PAGE using GRSF1, FASTKD4 and PNPT1 antibodies.

We and others have previously shown that FASTKD4 is required for the processing of the non-canonical *MT-ND5:MT-CYTB* RNA junction in the mitochondrial transcriptome (9,10), however, the exact role of FASTKD4 has remained unknown. Here, we showed that *MT-ND3* mRNA expression was decreased in the absence of FASTKD4 and that, in addition, its size was also slightly decreased. We then investigated the effects of chloramphenicol in the presence and absence of FASTKD4 and revealed that mitoribosome stalling recovered the stability of *MT-ND3* mRNA in the absence of FASTKD4 (Figure 2A), suggesting that FASTKD4 is required for the translation-dependent stability of *MT-ND3* mRNA. However, addition of chloramphenicol did not restore the size of the *MT-ND3* mRNA. The *MT-ND5:MT-CYTB* RNA processing defect in the absence of FASTKD4 was not affected by the presence of chloramphenicol (Figure 2A), indicating that FASTKD4 has roles in non-canonical mtRNA processing, RNA stability and translation.

To identify which end of the *MT-ND3* mRNA is protected by FASTKD4, we hybridized single-stranded oligonucleotides to either the 5′ or 3′ end and induced cleavage of the DNA/RNA hybrid by RNase H (Figure 2B). The 3′ end of *MT-ND3* was shorter in *FASTKD4^−/−^* cells in denaturing agarose (Figure 2B) and polyacrylamide gels (Figure 2C), revealing that FASTKD4 stabilizes the 3′ end of *MT-ND3*. Since the 3′ end of *MT-ND3* is polyadenylated and the RBP LRPPRC:SLIRP complex binds the polyadenylated tails of mtRNAs (35–37), FASTKD4 was immunoprecipitated and we found that it associated with LRPPRC but only in the presence of mtRNA in WT cells, but not in rho° cells that lack mtDNA (Supplementary Figure S3A). LRPPRC did not associate with FASTKD4 in control cells treated with RNase A or in cells lacking FASTKD4 (Supplementary Figure S3B), further validating the RNA-dependent interaction between FASTKD4 and LRPPRC. Interestingly, FASTKD4 did not associate with SLIRP, the binding partner of LRPPRC (Supplementary Figure S3C), further indicating that the interactions between LRPPRC and FASTKD4 is via RNA, since SLIRP does not bind mitochondrial RNAs in its association with LRPPRC (36).

Since we established that FASTKD4 associated in an RNA-dependent manner with LRPPRC and protected the 3′ end of *MT-ND3* mRNA, next we investigated the association of FASTKD4 in mitochondrial lysates with *in vitro* transcribed *MT-ND3* mRNAs that included diverse 3′ end tails that were tested for binding single- and double-stranded RNA (Figure 2C). We found that FASTKD4 was precipitated only with polyadenylated *MT-ND3* mRNA (Figure 2C), showing its specificity for the polyadenylated 3′ end of *MT-ND3* mRNA. In contrast, additional proteins used as controls, such as the polyribonucleotide nucleotidyltransferase 1 (PNPT) or the G-rich RNA sequence binding factor 1 (GRSF1), associated with all *MT-ND3* transcripts or all double stranded transcripts, respectively (Figure 2C and Supplementary Figure S3C), further indicating the specificity of FASTKD4 for the polyadenylated 3′ end of *MT-ND3* mRNA, which was also validated by an electrophoretic mobility shift assay (Supplementary Figure S3D).

### The mechanism of FASTKD4 action on *MT-ND3* mRNA

**Figure 3.**
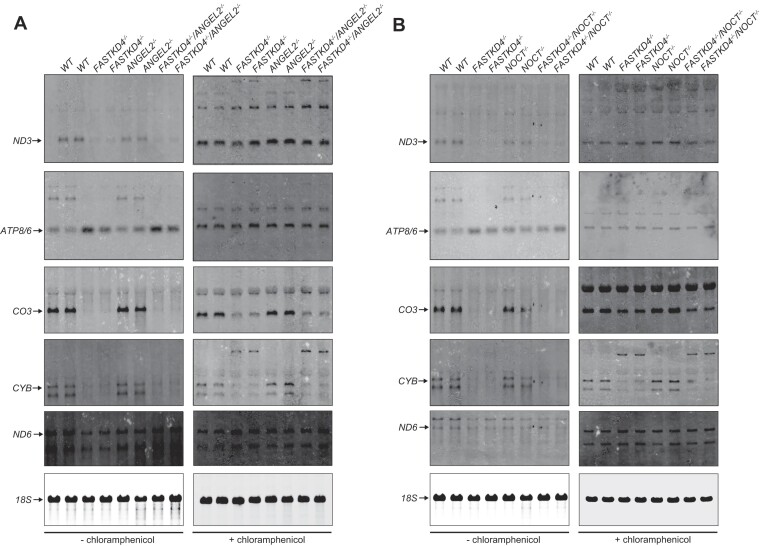
Inhibition of translation protects *MT-ND3* mRNA from degradation in the absence of FASTKD4. Detection of RNA levels via northern blotting in untreated or treated WT, *FASTKD4* knockout (*D4^−/−^*), *ANGEL2* single or double knockout (**A**) or *Nocturin* single or double knockout (*NOCT^−/−^*) cells (**B**). Cells were either treated with 20 μg/ml chloramphenicol or untreated and 10 μg isolated RNA was used for northern blotting and probed for *MT-ND3*, *MT-ATP8/6*, *MT-CO3*, *MT-CYB*, *MT-ND6* mRNAs and 18S rRNA. Representative blots are shown of three independently run biological experiments.

Since FASTKD4 bound the 3′ end of *MT-ND3* mRNA, we investigated the roles of two RNases that can affect the 3′ ends of mRNAs. ANGEL2 excises the 3′ phosphates of mt-mRNAs that are produced by non-canonical RNA processing (38), thereby enabling the polyadenylation of their ends by the mitochondrial poly(A) polymerase (38–40). The *Drosophila* homolog of FASTKD4 was found associated with ANGEL2 and its loss prevented ANGEL2 hydrolysis and processing of the non-canonical mt-mRNAs (38). We used northern blotting to investigate the 3′ end effects of ANGEL2 and FASTKD4 loss on the stability of diverse mt-mRNAs including *MT-ND3* in HAP1 cells (Figure 3A). In the absence of FASTKD4 there was a specific loss of *MT-ND3*, *MT-CO3*, *MT-CYB* and reduction in *MT-ND6* mRNAs, whereas loss of ANGEL2 reduced the levels of *MT-ND3* only very slightly (Figure 3A) under normal conditions. Double knockout of FASTKD4 and ANGEL2 resulted in the same effects as FASTKD4 only deletion (Figure 3A). Chloramphenicol treatment of the cells increased the stability of the affected mt-mRNAs and restored the stability of *MT-ND3, MT-CO3* and *MT-ND6* mRNAs to those of control cells (Figure 3A) in the *FASTKD4^−/−^* and double knockout (DKO) cells, confirming that stalled mitochondrial ribosomes can protect the ends of *MT-ND3* mRNA from degradation in the absence of FASTKD4, as seen in Figure 2A.

Next, we investigated the effects of the exonuclease Nocturnin, which is most closely related to a family of CCR4 deadenylases that remove polyadenylation from mRNAs, and localises to the cytoplasm and mitochondria (41,42). Loss of Nocturnin only reduced the stability of *MT-ND3* mRNA and the double knockout of Nocturnin and FASTKD4 resulted in the same defects as those caused by FASTKD4 loss alone (Figure 3B). Chloramphenicol treatment restored the stability of *MT-ND3* mRNA to that of control cells in the *FASTKD4^−/−^* and *FASTKD4^−/−^*/*NOCT^−/−^* DKO cells (Figure 3B), further confirming the protective effects of inhibited mitochondrial translation on *MT-ND3* mRNA.

**Figure 4.**
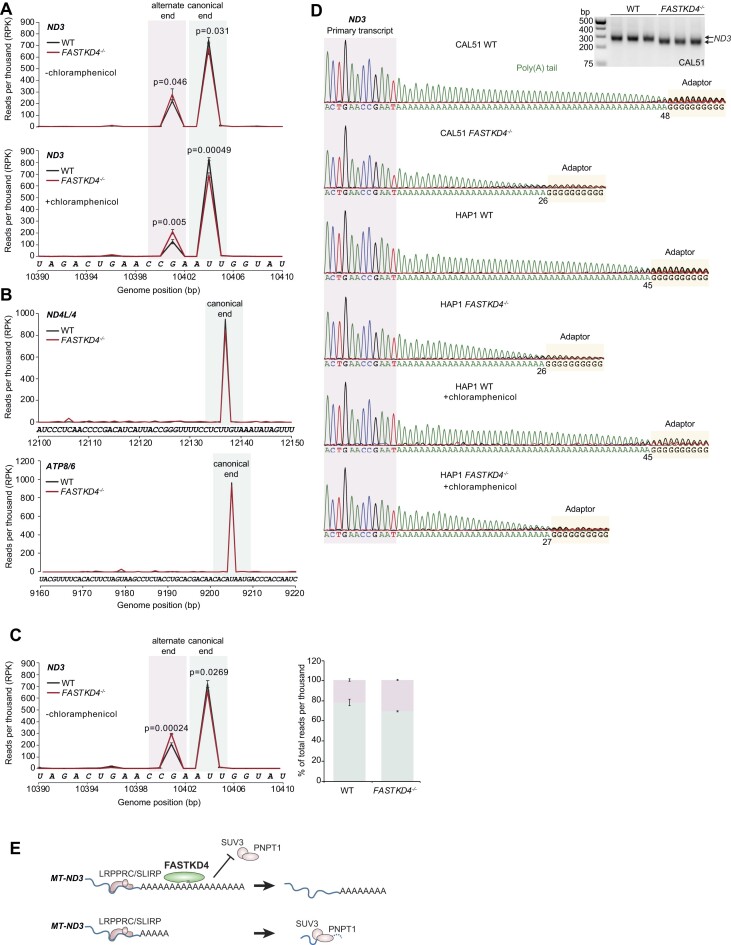
FASTKD4 protects the polyadenylated end of *MT-ND3* required for the stability of this mRNA. (**A**) Levels of alternate and canonical *MT-ND3* mRNA ends or (**B**) *MT-ND4L/4* and *MT-ATP8/6* mRNA ends in HAP1 cells or in CAL51 cells (**C**) were measured by poly(A) capture and next generation sequencing. (**D**) Comparison of polyadenylation of *MT-ND3* RNA ends. WT or *FASTKD4^−/−^* cells were either untreated or treated with 20 μg/mL chloramphenicol prior to poly(A) capture and Sanger sequencing. Three biologically independent samples were used in each experiment in Figure 4, the results are mean ± SD (Student's t test, two-tailed unpaired t-test) and *P* values are shown. (**E**) Model of FASTKD4 function, where FASTKD4 protects the length of the poly(A) tail from the *MT-ND3* mRNA, and in its absence there is reduced polyadenylation of this mRNA, compromising its stability.

To test whether FASTKD4 loss had an effect on the *MT-ND3* RNA poly(A) tail, we ligated adapters to accurately capture the 3′ ends of mt-mRNAs by sequencing. Unexpectedly, we identified that *MT-ND3* mRNA has a canonical 3′ end and an alternate 3′ end that lacks the final three nucleotides including the last U, precluding the formation of a stop codon by polyadenylation (Figure 4A). The *MT-ND3* mRNA with the canonical 3′ end was reduced significantly in the *FASTKD4^−/−^* cells whereas *MT-ND3* mRNA with the alternate 3′ end was increased (Figure 4A), indicating that FASTKD4 is required for the stability of *MT-ND3* mRNA with the canonical 3′ end. Chloramphenicol treatment reduced the levels of the *MT-ND3* mRNA with the alternate 3′ end (Figure 4A). The 3′ ends of *MT-ND4L/4* and *MT-ATP8/6* mRNAs were not affected by the loss of FASTKD4 and they maintained their canonical ends (Figure 4B). These results were consistent in both, control and FASTKD4 knockout CAL51 breast cancer cells, which are diploid cell lines, validating that these findings are not a consequence of the haploid status of the HAP1 cells (Figure 4C).

Next, we measured the poly(A) tails of *MT-ND3* mRNA and found that in the absence of FASTKD4, the polyadenylation of *MT-ND3* was decreased compared to control cells (Figure 4D). The decrease in poly(A) tail length likely explains the reduced size of the *MT-ND3* mRNA seen in northern blots in the absence of FASTKD4, and this was confirmed by RT-PCR of the 3′ ends of *MT-ND3* (Figure 4D). These findings suggest that FASTKD4 binding at the polyadenylated 3′ end of *MT-ND3* mRNA, through its RNA-dependent association with LRPPRC (Supplementary Figure S3B), protects its level of polyadenylation. FASTKD4 prevents degradation of *MT-ND3* mRNA by binding its polyadenylated tail, since loss of FASTKD4 reduces the stability of this transcript, and further reduction of PNP1 and Suv3 increase its stability specifically, compared to reduction of other mitochondrial RNA-regulating factors (Supplementary Figure S4). Reduction of polyadenylation in the FASTKD4 knockout CAL51 breast cancer cells compared to control CAL51 cells confirmed that the molecular role of FASTKD4 was consistent with the HAP1 cells (Figure 4D). In the absence of FASTKD4, chloramphenicol treatment the polyadenylation of *MT-ND3* remained reduced (Figure 4D). Since chloramphenicol treatment could not restore the level of polyadenylation in the FASTKD4 knockout cells (Figure 4D), this confirms that reduced polyadenylation of *MT-ND3* in the absence of FASTKD4 is independent of translation. Therefore, the binding of FASTKD4 is required to protect the *MT-ND3* poly(A) tail from degradation and maintain the stability of this mRNA in mitochondria (Figure 4E).

## Discussion

Energy conversion for cellular function is dependent on the harmonized regulation of mitochondrial and nuclear gene expression (1,43). The mitochondrial transcriptome is regulated by RBPs, which play roles from transcription to degradation (1). Recently, a number of RNA modifying enzymes and RNA-binding adapter proteins have been identified, that are required to fine-tune the expression of the mitochondrial genome, particularly to accommodate its unusual features, such as non-canonical RNA processing (38,44,45). The FASTK family of RBPs has been identified as important modulators of mitochondrial gene expression that affect specific and discrete functions, including non-canonical RNA processing, stability of the only non-polyadenylated mRNA, *MT-ND6*, and protein synthesis (8–10,13,46). Here we show that FASTKD4, as well as having a role in non-canonical RNA processing, is required for the stability of the *MT-ND3* mRNA through its association with the LRPPRC:SLIRP complex at the 3′ end to protect its polyadenylated tail from degradation. The loss of FASTD4 resulted in a shorter and hence, faster migrating *MT-ND3* mRNA, a phenomenon that was consistent in all tested cell types. We identified that the *MT-ND3* mRNA has both a canonical and alternate end, and that in the absence of FASTKD4 the polyadenylation of *MT-ND3* mRNA is reduced, leading to its instability. The *MT-ND3* mRNA with the alternate 3′ end lacks its stop codon and therefore is likely degraded when it is recognized as a non-stop mRNA by translating mitoribosomes. Therefore, FASTKD4 is a *MT-ND3* mRNA-specific factor that ensures the poly(A) tail is maintained to secure its stability.

Mutagenesis of key residues based on the molecular structure of the RNA-binding domains of FASTKD4 revealed that the central ‘Lock’ and C-terminal ‘Key’ helices are important for both its stability and function. The full-length protein could not be produced effectively as it resulted in aggregation and based on our structure, we speculate that it may be possible for the ‘Lock’ helix to be displaced by insertion of the ‘Key’ helix (E610-L632) to promote tandem oligomerization. Blocking the insertion by stabilizing the central ‘Lock’ helix may constrain the protein in a monomeric form that could potentially improve the stability of the aggregation-prone full-length protein. Previously it was suggested that FASTKD4 may have a nuclease activity based on a prediction that the D531 residue formed a putative active site of FASTKD4 (34). In that work a D531A mutation was unable to rescue the processing of *ND5-CYB* precursor RNA in *FASTKD4^−/−^* cells (34). Our atomic structure of FASTKD4 now reveales a cavity with close similarity to the active site of the Vsr endonuclease, which suggested that residue D523 may be a putative catalytic site, however, mutation of this site in FASTKD4 did not alter the stability of *MT-ND3*. This suggests that FASTKD4 does not have nuclease activity but has adapted to the requirements of the minimal mitochondrial transcriptome to fine-tune the maturation and polyadenylation of a specific mRNA by direct binding, without cleavage, to ensure its integrity for translation.

The role of FASTKD4 increases the repertoire of adapter or scaffold RBPs required by specific mitochondrial RNAs to support their stability or modification, such as the LRPPRC/SLIRP complex, FASTK and PTCD1 (36,45,46). Although FASTKD4 has lost its nuclease activity, there are active site enzymes such as TRMT10C and SDR5C1, which act as scaffolds to support the endonuclease activities of the tRNA processing enyzmes PRORP and ELAC2 (47–49). It is possible that FASTKD4 structurally supports the recently discovered nuclease activity of FASTKD5 (50), since loss of FASTKD4 impairs the processing of *MT-ND5* RNA in a similar way as loss of FASTKD5. The compaction of animal mitochondrial genomes over evolution and the unique features of mitochondrial transcripts has necessitated the adoption and repurposing of RBPs to regulate their expression and stability, opening the scope of mitochondrial gene expression for the discovery of additional transcript-specific factors.

## Supplementary Material

gkae1261_Supplemental_File

## Data Availability

Crystallographic coordinates and associated structure factors have been deposited with the Protein Data Bank (http://rcsb.org) with accession number 9GEK. MiSeq data have been deposited in the SRA database with accession number PRJNA1178748.
