# Supplementary material for: The Vsr-like protein FASTKD4 regulates the stability and polyadenylation of the MT-ND3 mRNA
Source: Nucleic Acids Res. 2024 Dec 27;53(4):gkae1261. doi: 10.1093/nar/gkae1261 (PMC11879112; doi:10.1093/nar/gkae1261)
Supplement: gkae1261_Supplemental_File [file gkae1261_supplemental_file.docx]

**The Vsr-like protein FASTKD4 regulates the stability and polyadenylation of the *MT-ND3* mRNA**

Xuan Yang^1#^, Maike Stentenbach^2,3#^, Laetitia A. Hughes^2,3#^, Stefan J. Siira^2,3^, Kelvin Lau^4^, Michael Hothorn^4^, Jean-Claude Martinou^1*^, Oliver Rackham^2,3,5,6*^, and Aleksandra Filipovska^2,3,5*^

^1^Department of Molecular Cell Biology, University of Geneva, 1211 Geneva, Switzerland

^2^The Kids Research Institute Australia, Northern Entrance, Perth Children's Hospital, 15 Hospital Avenue and ^3^ARC Centre of Excellence in Synthetic Biology, Nedlands, Western Australia 6009, Australia

^4^Department of Plant Sciences, University of Geneva, 1211 Geneva, Switzerland

^5^Curtin Medical School, Curtin University, Bentley, Western Australia 6102, Australia

^6^Curtin Health Innovation Research Institute, Curtin University, Bentley, Western Australia 6102, Australia

**Contents**

**Supplementary Figure 1.** Generation of *FASTKD4* mutants and structural comparison to other endonuclease domains.

**Supplementary Figure 2.** Conservation of FASTKD4-related protein sequences corresponding to the human FASTKD4 region determined by crystallography.

**Supplementary Figure 3.** Interaction of FASTKD4 with LRPPRC in an RNA-dependant manner.

**Supplementary Figure 4.** Effects of siRNA treatment on *MT-ND3* mRNA levels.

**Supplementary Table 1.** Crystallographic data collection and refinement statistics.


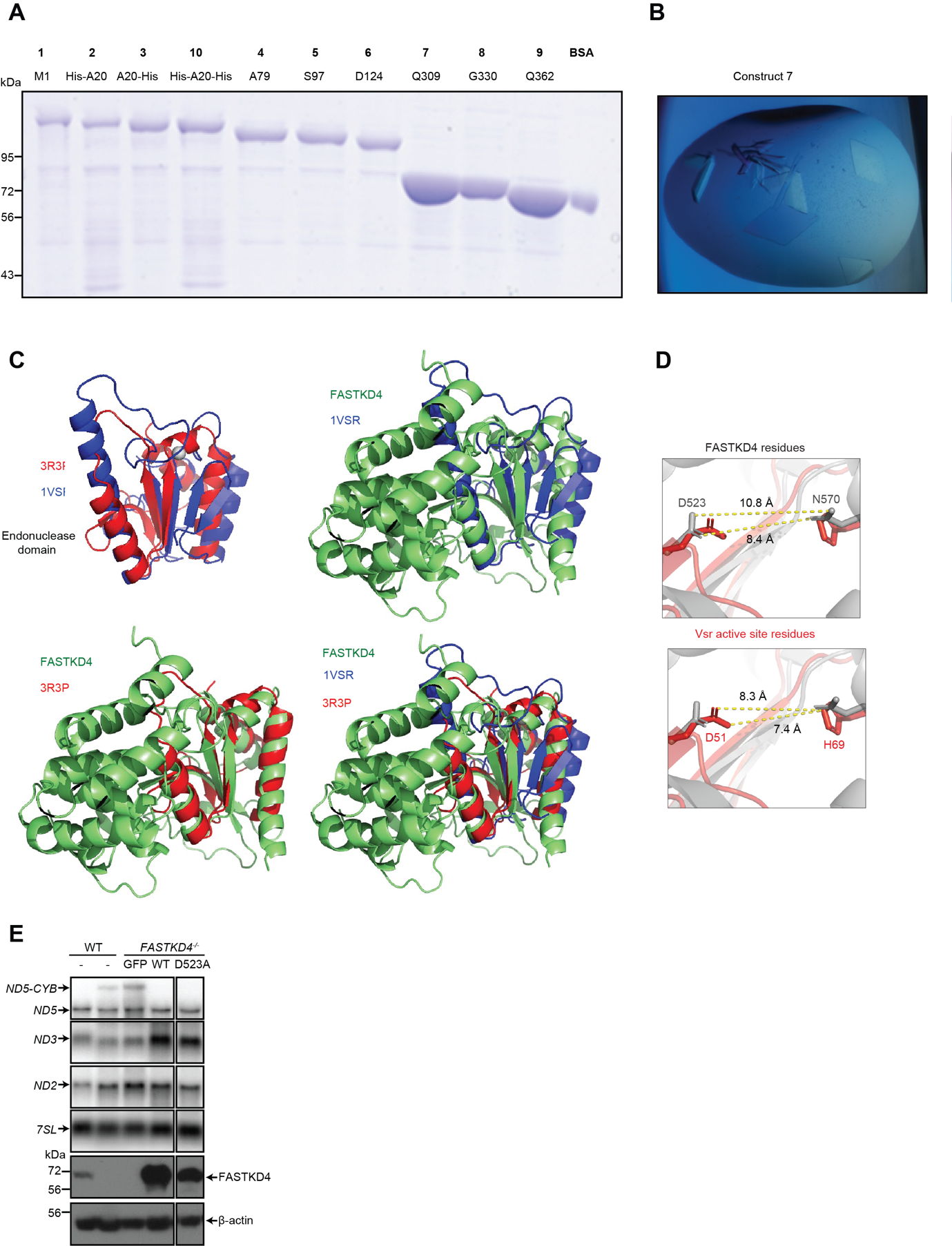


**Supplementary Figure 1. Generation of *FASTKD4* mutants and structural comparison to other endonuclease domains.** (**A**) Expression and purification of *FASTKD4* mutants​. (**B**) Crystals of the RNA-binding domains of FASTKD4 construct 7. (**C**) Superposition of the active sites of 1VSR, 3R3P with the positively charged cleft identified in FASTKD4​. (**D**) Superposition of the FASTKD4 cleft residues and Vsr active site residues with labelled interside-chain distances. (**E**) Detection of RNA and protein levels in Control (WT) and *FASTKD4*^-/-^ cells expressing GFP, control (WT) or the FASTKD4 D523A mutant protein. 6 µg RNA or 20 µg protein was separated by size via electrophoresis or SDS-PAGE respectively, transferred to a respective membrane and probed for *MT-ND5, MT-ND3, MT-ND2* and *7SL* RNAs by northern blotting of probed for FASTKD4, and β-actin (used as a loading control) by immunoblotting.

**
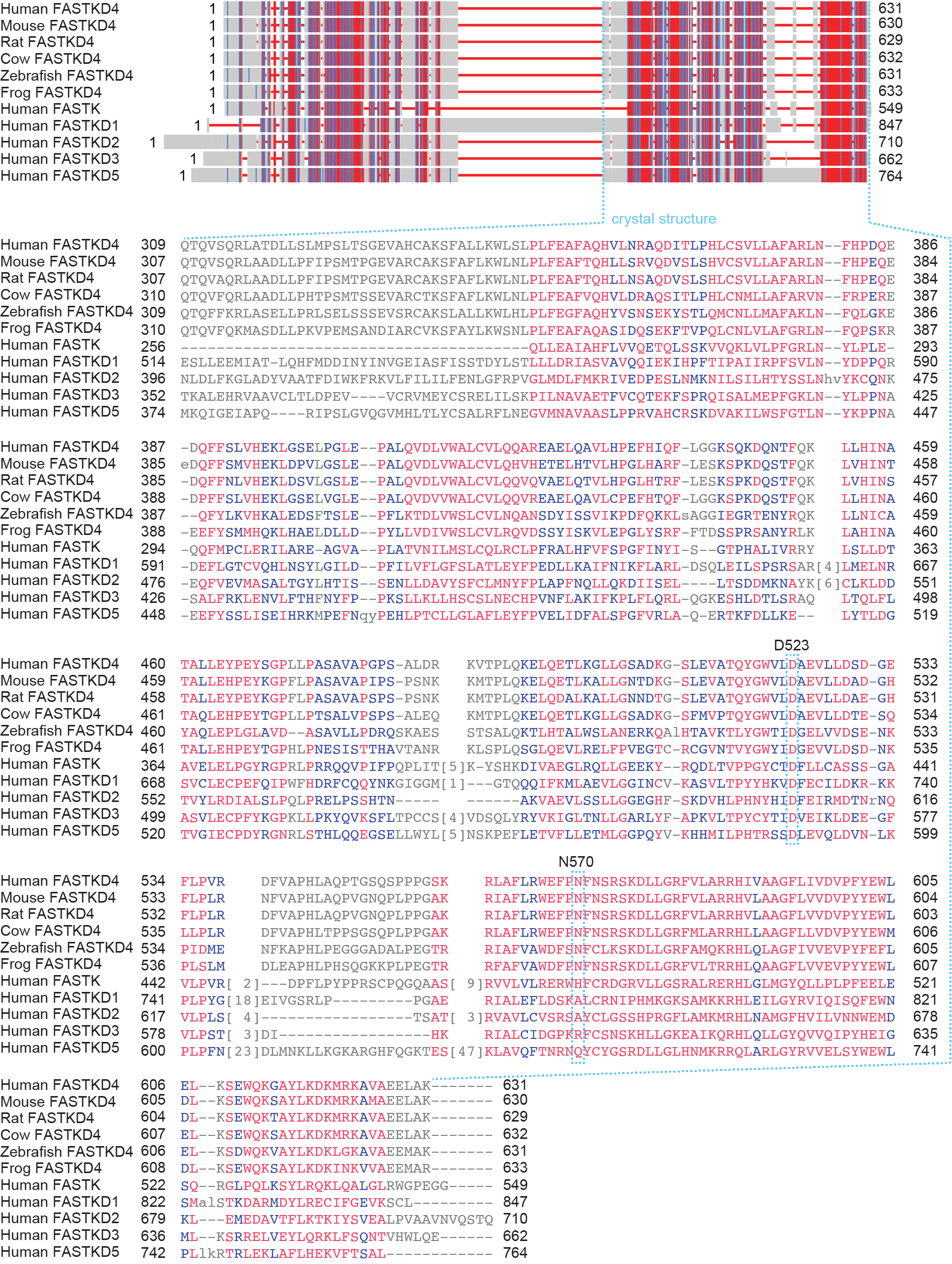
**

**Supplementary Figure 2.** Conservation of FASTKD4-related protein sequences corresponding to the human FASTKD4 region determined by crystallography. Highly conserved amino acids are coloured in red (2 Bit conservation setting), and dark blue indicates less conserved amino acids. Residues corresponding to D523 and N570 in human FASTKD4 are boxed in light blue. Sequences used were obtained from GenBank at NCBI (human FASTKD4, *Homo sapiens*, NP_004740.2; mouse FASTKD4, *Mus musculus*, NP_598772.1; rat FASTKD4, *Rattus norvegicus*, NP_001012154.1; cow FASTKD4, *Bos taurus*, NP_001070435.1; zebrafish FASTKD4, *Danio rerio*, XP_690863.4; frog FASTKD4, *Xenopus tropicalis*, NP_001004904.2; human FASTK, *Homo sapiens*, NP_006703.1; human FASTKD1, *Homo sapiens*, NP_001308975.1; human FASTKD2, *Homo sapiens*, NP_001129666.1; human FASTKD3, *Homo sapiens*, NP_076996.2; human FASTKD5, *Homo sapiens*, NP_068598.1) and the alignment was produced using COBALT (NCBI).


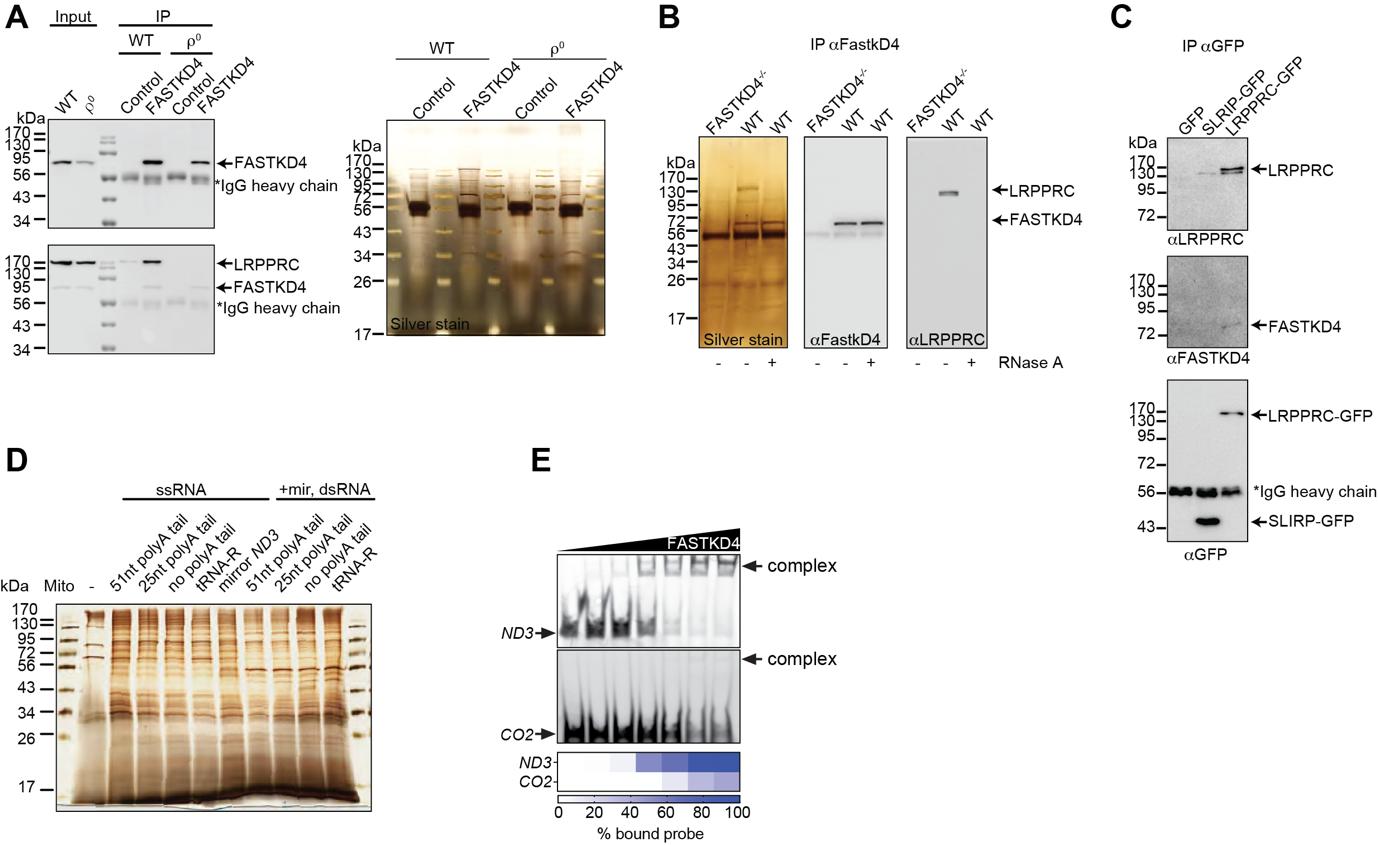


**Supplementary Figure 3. Interaction of FASTKD4 with LRPPRC in RNA dependant matter.** (**A**) Immunoprecipitation of FASTKD4 and its interaction with LRPPRC in the presence or absence of mtRNA in 143B and 143B rho zero cells, respectively was detected by immunoblotting for either FASTKD4 or LRPPRC. (**B**) Immunoprecipitation of FASTKD4 and its interaction with LRPPRC in the presence or absence of mtRNA. Effects of RNase A treatment on LRPPRC/FASKD4 interaction. 143B cells were transfected with GFP-flagged FASTKD4 and immunoprecipitated. Protein interaction was confirmed through western blotting in the presence or absence of mtRNA or after RNase A treatment respectively. Equal protein loading was confirmed through silver stain. (**C**) 143B cells were transfected with GFP-flagged LRPPRC or SLIRP, or GFP alone as a control and immunoprecipitated using a GFP antibody. Immunoprecipitation was detected using a LRPPRC or FASTDK4 antibodies. The expression of the GFP-bait proteins was confirmed by western blotting against the GFP tag of the proteins. (**D**) Silver-stained loading control for the *in vitro* binding reactions of *MT-ND3* shown in the main Figure 2C. HEK293T mitochondria were incubated 4 µg of the following IVT RNA: 51 nt polyA tail, 25 nt polyA tail, without polyA tail, tRNA-R or mirror *ND3* RNA. (**E**) RNA electrophoretic mobility shift assay showing greater binding affinity of FASTKD4 for the *ND3* mRNA compared to the *CO2* mRNA. Serial dilution of FASTKD4 was incubated with fluorescently labelled *ND3* or *CO2* RNAs containing the 3' end and poly(A) tail of each mRNA for 30 minutes and the complexes were resolved by electrophoresis on a 6% native gel.

**
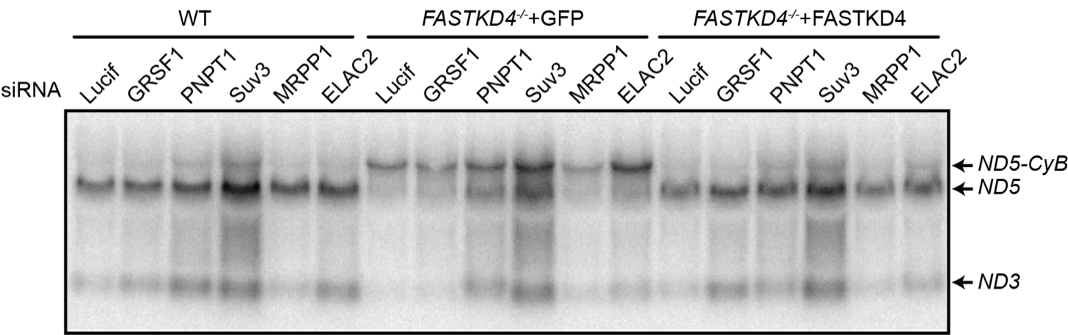
**

**Supplementary Figure 4. Effects of siRNA treatment on *MT-ND3* levels.** (**A**) Northern blots of wild-type (WT) and *FASTKD4^-/-^* cells after silencing of luciferase, GRSF1, PNPT1, Suv3, MRPP1 or ELAC2. Cells were incubated with 10 nM siRNA for 72 hours prior to RNA extraction. 10 µg RNA was used for northern blotting and probed for *MT-ND3* and *MT-ND5* mRNAs.

**Supplementary Table 1.** Crystallographic data collection and refinement statistics.

|  | **MBP-FASTKD4^309_631^** |
| --- | --- |
| **PDB-ID** | **9GEK** |
| **Data collection** |  |
| Wavelength | 1.000027 |
| Space group | *P* 2 2_1_ 2_1_ |
| Cell dimensions |  |
| *a*, *b*, *c* (Å) | 80.06, 80.06, 114.81 |
| α, β, γ (°) | 90, 90, 90 |
| Resolution (Å) | 48.13 – 2.15 (2.28 – 2.15) |
| *R_meas_^#^* | 0.19 (2.15) |
| CC(1/2)*^#^* | 1.0 (0.36) |
| *I/σ I^#^* | 10.9 (1.1) |
| Completeness (%)*^#^* | 99.3 (98.2) |
| Redundancy*^#^* | 11.2 (9.0) |
| Wilson B-factor*^#^* | 28.9 |
| **Refinement** |  |
| Resolution (Å) | 48.13 – 2.15 |
| No. reflections | 85,063 |
| *R*_work_ / *R*_free_^+^ | 0.20 / 0.24 |
| No. atoms |  |
| protein | 5,368 |
| ligand | 73 |
| solvent | 261 |
| Res. B-factors^+^ |  |
| protein | 46.9 |
| ligand | 64.8 |
| solvent | 41.4 |
| R.m.s deviations^$^ |  |
| bond lengths (Å) | 0.021 |
| bond angles (°) | 0.54 |
| Ramachandran plot^$^: |  |
| most favored regions (%) | 98.1 |
| outliers (%) | 0 |
| MolProbity score^$^ | 1.0 |

^#^as defined in XDS (Kabsch, 1993)

^+^as defined in phenix.refine (Afonine *et al*, 2012)

^$^as defined in Molprobity (Davis *et al*, 2007b)

**Supplementary references:**

Afonine PV, Grosse-Kunstleve RW, Echols N, Headd JJ, Moriarty NW, Mustyakimov M, Terwilliger TC, Urzhumtsev A, Zwart PH & Adams PD (2012) Towards automated crystallographic structure refinement with phenix.refine. *Acta Cryst D* 68: 352–367

Davis IW, Leaver-Fay A, Chen VB, Block JN, Kapral GJ, Wang X, Murray LW, Arendall WB, Snoeyink J, Richardson JS, *et al* (2007) MolProbity: all-atom contacts and structure validation for proteins and nucleic acids. *Nucleic Acids Res* 35: W375-383

Kabsch W (1993) Automatic processing of rotation diffraction data from crystals of initially unknown symmetry land cell constants. *Journal of Applied Crystallography* 26:795-800.
